# Levels of handwashing and vaccine uptake in Kenya, Uganda, and Tanzania to prevent and control COVID-19: a systematic review and meta-analysis

**DOI:** 10.3389/fpubh.2023.1256007

**Published:** 2023-11-09

**Authors:** Josphat Martin Muchangi, James Mturi, Hajra Mukasa, Kioko Kithuki, Sarah Jebet Kosgei, Lennah Muhoja Kanyangi, Rogers Mararo, Maureen Nankanja

**Affiliations:** ^1^Amref Health Africa, Nairobi, Kenya; ^2^Amref Health Africa, Dar es Salaam, Tanzania; ^3^Amref Health Africa, Kampala, Uganda

**Keywords:** handwashing, vaccine, COVID-19, prevention, control, policy, Kenya, Uganda

## Abstract

**Introduction:**

Coronavirus Disease 2019 (COVID-19) presents a massive challenge in Africa due to overwhelmed and underresourced health systems, as well as the existing burden of communicable and non- communicable diseases. Self-inoculation may occur when an individual touches their mucous membrane following direct contact between their hands and severe acute respiratory syndrome coronavirus-2 (SARS-CoV-2)-contaminated surfaces. Therefore, handwashing can be used along with COVID-19 vaccines to reduce the spread of SARS-CoV-2 and the burden of COVID-19. We were interested in investigating the levels of handwashing and vaccine uptake to control and prevent COVID-19 in Kenya, Uganda and Tanzania using a systematic review and meta-analysis.

**Methods:**

We searched PubMed, African Index Medicus and African Journals Online for studies published since inception to 31^st^ January 2023. We included all studies that assessed: the levels of COVID-19 vaccine acceptance and hesitance as indicators of vaccine uptake; and studies on the levels of handwashing to prevent and control COVID-19 in Kenya, Uganda and Tanzania. Study findings were synthesized by meta-analysis to get the pooled effect measure. Three studies were synthesized qualitatively due to high level of heterogeneity in effect measure precluding a quantitative meta-analysis.

**Results:**

Our search identified 128 articles of which 17 studies with 18,305 participants and 622 vaccination sites were reviewed with 14 of them being meta-analyzed. This systematic review and meta-analysis reports high levels of COVID-19 vaccine acceptance and handwashing in Kenya, Uganda and Tanzania at 67% (95% CI: 55, 78) and 88% (95% CI: 73, 97), respectively. Vaccine hesitance among the participants was low at 31% (95% CI: 15, 49).

**Discussion:**

Despite their importance in the control and prevention of COVID-19, some countries do not implement handwashing and vaccination effectively. There is a need for public health strategies to raise awareness about the importance of handwashing and the uptake of the COVID-19 vaccine.

**Systematic Review Registration:**

https://www.crd.york.ac.uk/PROSPERO/, PROSPERO ID CRD42023394698.

## Introduction

Severe acute respiratory syndrome coronavirus 2 (SARS-CoV-2), the causal agent of Coronavirus Disease 2019 (COVID-19), is transmitted via respiratory droplets (1). Self-inoculation may occur when one touches their mucous membranes of the nose, mouth, or eyes after direct contact with contaminated surfaces (2). Further, SARS-CoV-2 can be transmitted indirectly when contaminated hands spread the virus to other surfaces (2). As of 16^th^ March 2023, the World Health Organization (WHO) had reported over 760 million verified cases of COVID-19 including more than six million deaths (3). Coronavirus Disease 2019 (COVID-19) presents a massive challenge in Africa due to overwhelmed and underresourced health systems, as well as the existing burden of communicable and non- communicable diseases (4).

Studies have reported that handwashing is beneficial in preventing a range of infections, including respiratory infections, gastrointestinal illnesses, and soil helminth infections (5–7). The WHO reported handwashing with soap and water as an effective and affordable way to prevent the transmission of SARS-CoV-2 (8). Similarly, studies show that handwashing reduces chances of self-contamination and subsequent nasal inoculation (8–10). According to a study, the practice of handwashing was found to reduce the transmission of respiratory viruses by approximately 45–55% (11). In addition, Saunders-Hastings et al. reported that hand hygiene decreased the spread of H1N1 influenza in humans by 38% (12). Evaluation of adult participants showed that handwashing was effectual in decreasing the spread of influenza virus (13). Despite the critical need for working handwashing stations at community and structural levels, it was estimated that the majority of people living in sub-Saharan Africa lacked access to handwashing stations before the COVID-19 pandemic (14).

Vaccination prevents SARS-CoV-2 infection and lowers the risk of severe health outcomes linked to COVID-19 (15, 16). Clinical trials along with observational studies have reported numerous COVID-19 vaccines to be harmless and efficacious in averting severe illness and risk of death (17–19). More than 13 billion vaccine doses have been administered globally to control and prevent COVID-19 (3). It is important to achieve high coverage of COVID-19 vaccination to reduce the adverse economic and health impacts associated with the pandemic. Furthermore, in the face of new variants, booster doses as well as vaccines with updated formulations may be required (20).

Implementing public health and social measures such as handwashing and vaccination against COVID-19 can slow down the spread of SARS-CoV-2 (21). However, few studies have investigated the link between handwashing and vaccine uptake to control and prevent COVID-19 in East Africa and their findings are inconclusive. Therefore, our objective was to conduct a systematic review and meta-analysis of levels of handwashing and COVID-19 vaccine uptake in Kenya, Uganda and Tanzania to prevent and control COVID-19. The outcomes of interest included vaccine hesitance and vaccine acceptance as indicators of levels of vaccine uptake, and levels of handwashing. Consequently, this research seeks to address the following fundamental research question: In residents of Kenya, Uganda, and Tanzania, what is the prevalence of handwashing practices, vaccine hesitancy, and vaccine acceptance in the context of COVID-19 prevention and control?

## Materials and methods

### Study design

We conducted this study according to the Preferred Reporting Items for Systematic Reviews and Meta-Analyses (PRISMA) and the Centre for Reviews and Dissemination (CRD) guidelines (22, 23). The systematic review and meta-analysis was registered under registration number CRD42023394698 on the International Prospective Register of Systematic Reviews (PROSPERO) database.

### Eligibility criteria

*Inclusion criteria*: (1) studies on the levels of COVID-19 vaccine acceptance as an indicator of vaccine uptake in Kenya, Uganda and Tanzania; (2) studies on the levels of COVID-19 vaccine hesitance as an indicator of vaccine uptake in Kenya, Uganda and Tanzania; (3) studies on the levels of handwashing to prevent and control COVID-19 in Kenya, Uganda and Tanzania; (4) studies published in any language were considered for inclusion.

*Exclusion criteria*: (1) literature reviews, conference abstracts, and case series; (2) preprints; (3) articles with unclear measures of vaccine uptake; (4) studies conducted in countries other than Kenya, Uganda and Tanzania; (4) studies on COVID-19 vaccine side effects; (5) studies without the relevant exposure or treatment were excluded.

### Literature search

We conducted a systematic literature search in PubMed, African Index Medicus and African Journals Online to select plausibly eligible articles published since database inception to 31^st^ January 2023. Moreover, we manually screened citations of eligible articles to identify additional studies. We formulated a search strategy based on the PECOS framework by combining the terms handwashing, vaccine, COVID-19, prevention, policy, Kenya, Uganda, and Tanzania. The search approach used in the PubMed database was modified to suit other databases. The detailed approach of literature search is available in Supplementary Tables 1, 2.

### Study selection

We used the Mendeley reference manager to manage the articles identified during the search. Identical studies were initially excluded using Mendeley after which irrelevant articles were removed by screening the titles and abstracts for relevance. We then reviewed full texts of the potentially relevant studies to determine eligibility for inclusion. The eligible studies were selected by two independent reviewers and discordant outcomes were resolved through discussion.

### Data extraction

Two independent researchers extracted data from the eligible articles using a predefined and standardized excel sheet. Variables that were extracted from these studies included: (1) the name of the first author(s); (2) the title of the study; (3) the year of publication; (4) study objective(s); (5) the publishing journal; (6) the study design; (7) the sample size; (8) participants’ characteristics including age; (9) the inclusion and exclusion criteria; (10) indicators of hand washing levels; (11) indicators of vaccine uptake levels; (12) main finding and other findings. Missing data were obtained by contacting authors of the eligible studies.

### Quality assessment

We assessed the potential for bias in the eligible articles based on the Quality Assessment Tool for Observational Cohort and Cross-sectional studies (24).^1^ This checklist encompasses 14 crucial criteria regarded as fundamental for ensuring the quality of reporting in cohort and cross-sectional studies. These recommendations focus on various aspects, including the article’s objectives, the study population, exposure measures and potential confounders, among others.

### Statistical analysis

We conducted meta-analyses of single proportions to calculate the overall proportion using the metaprop function of the Meta package in R (version 4.1.2). Heterogeneity between studies was assessed using I^2^ statistics, with an I^2^ of more than 75% indicating substantial heterogeneity. We used a funnel plot to check for publication bias and the Eggers test for assessing funnel plot asymmetry. We implemented random effect meta-analysis because of the high levels of heterogeneity between study populations. Heterogeneous studies were synthesized through a narrative summary based on the specific outcome indicator.

## Results

### Study selection

**Figure 1:**
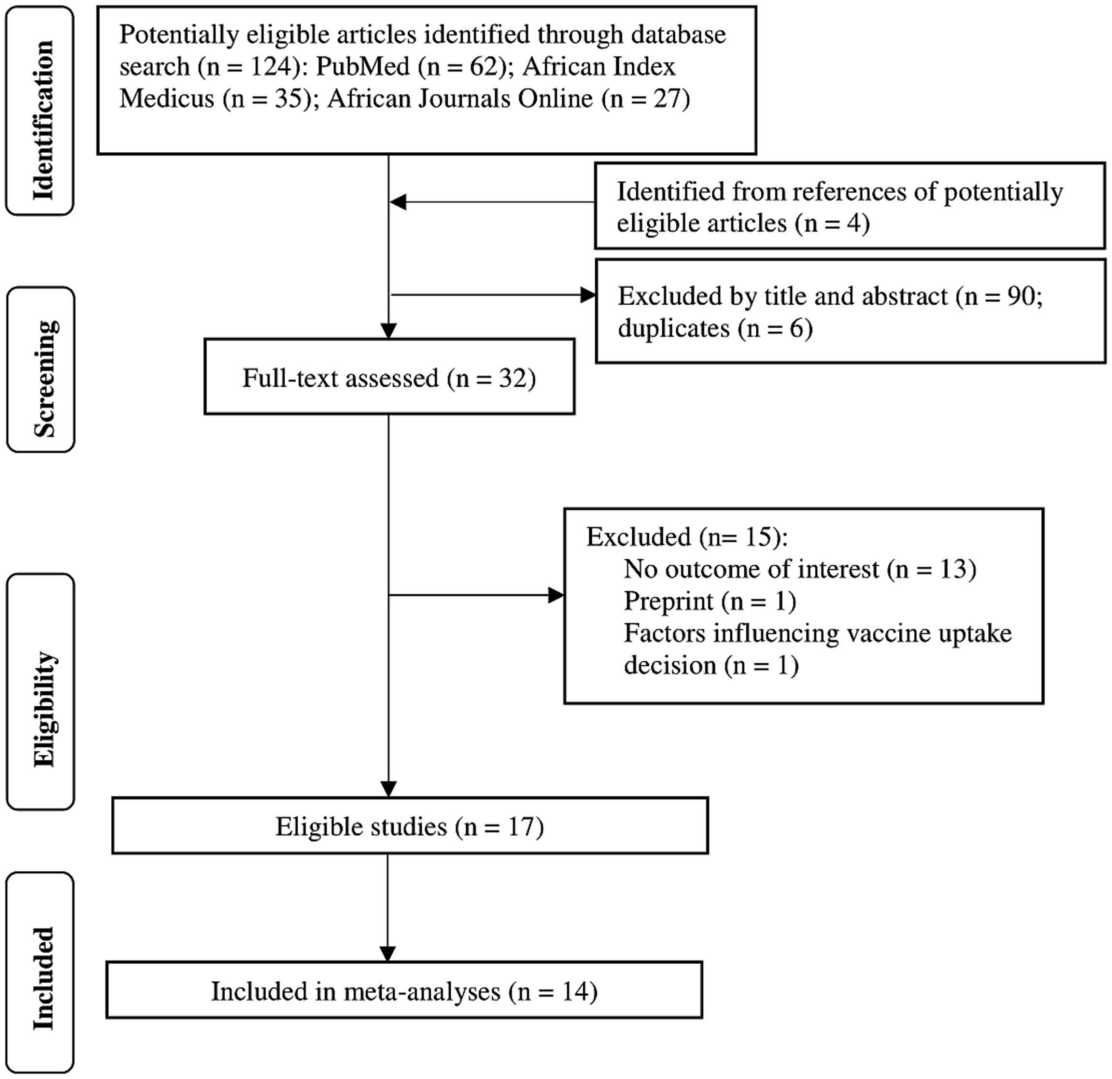
PRISMA chart depicting study selection process.

Our literature search yielded 124 articles and an additional four papers from potentially eligible articles (Figure 1). We excluded 6 duplicates and 90 studies that were irrelevant after screening titles and abstracts. We excluded 15 papers after a full-text review of the remaining articles, including one preprint and 14 others without the outcome of interest. A total of 17 studies with 18,305 participants and 622 vaccination sites were included in the systematic review. Only 14 of the 17 studies were meta-analyzed. Some studies could not be meta-analyzed because they reported heterogeneous effect estimates, including vaccination rates and odds ratios of getting the COVID-19 vaccine.

### Basic characteristics of eligible studies

We included observational studies published between 2021 and 2023 in this systematic review and meta-analysis. The eligible studies included 15 cross-sectional studies, one mixed-method study and one longitudinal study. Seven of these studies were conducted in Kenya, with Uganda and Tanzania having eight and two studies, respectively. The sample sizes varied across studies ranging from 33 to 4,136 participants. Only three of four studies that reported handwashing levels in Kenya, Uganda and Tanzania had handwashing as the primary outcome.

### Quality evaluation

According to the Quality Assessment Tool for Observational Cohort and Cross-sectional studies checklist, all the studies met the recommendations for conducting observational studies (Supplementary Table 3). This finding indicates high overall methodological quality and low risk of bias of these studies.

### Meta-analysis

#### Levels of handwashing in Kenya, Uganda and Tanzania

**Figure 2:**
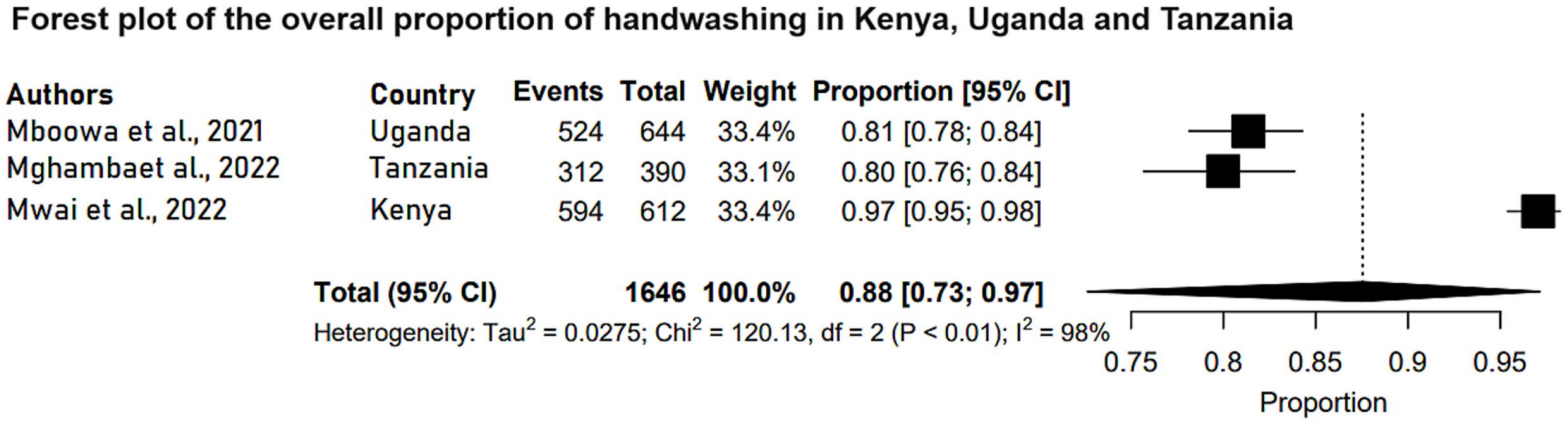
Forest plot of the overall proportion of handwashing in Kenya, Uganda and Tanzania. Each study is represented by a black box and a horizontal line, which correspond to the proportion and 95% confidence interval, respectively. *I*^2^ shows the degree of heterogeneity with value of *p* indicating whether there was statistically significance heterogeneity between the studies and among the groups.

The participants of the studies that reported handwashing levels as a way of preventing and controlling COVID-19 were adults with a mean (SD) ages ranging from 34.8(11.2) years to 38.2(14.8) years. These studies reported that approximately 80.4 to 97% of the respondents practiced handwashing and 28.6% of facilities enforced obligatory use of hand hygiene (Table 1). Further meta-analysis of three of those studies (N = 1,646) showed that the pooled estimate proportion was 0.88 (95% CI: 0.73, 0.97) (Figure 2).

**Table 1:** Levels of handwashing to prevent and control COVID-19 in Kenya, Uganda, and Tanzania.

First author, year	Country	Participants	Age mean(SD)	Sample size (male)	Study design	Dates of data collection	Outcome	Outcome definition	Main findings
Mghamba, 2022 (25)	Tanzania	Adults from five municipalities, namely Ilala, Ubungo, Kinondoni, Temeke and Kigamboni.	34.8(11.2) years	390 (194)	Cross-sectional study	April and May 2020	Levels of hand washing (1y)	The proportion of respondents practicing handwashing to prevent COVID-19	80.4% (*n* = 312) of the respondents reported that they implemented effective handwashing with water and soap or used alcohol-based gels/sanitizers.
Mboowa, 2021 (26)	Uganda	High-risk groups, namely food-market vendors, police officers and healthcare workers.	35.1(11.0) years	644 (340)	Cross-sectional study	July 2020	Handwashing (2y)	The proportion of participants practicing handwashing	81.4% of the participants practiced handwashing with soap and water for at least 20 s.
Okedi, 2022 (27)	Kenya	Medical officers, nursing officers, public health officers, registered clinical officers, laboratory technologists, pharmaceutical technologists, nurse attendants and subordinate staff of health facilities	NA	33 health facilities	Cross-sectional study	NA	Hand hygiene practices (1y)	Compliance with hand hygiene guidelines	Only 2 (28.6%.) of facilities enforced obligatory use of hand hygiene and there was no policy on hand hygiene in 6 (86%) health facilities.
Mwai, 2022 (28)	Kenya	Men and women who were household heads (18–60 years of age) and residing in Kilifi and Mombasa counties.	38.2(14.8) years	612 (181)	Cross-sectional survey	25 November and 3 December 2020	Hand hygiene practices (1y)	Practices of washing hands to control COVID-19	594 (97%) households indicated that they practice hand washing although 396 (64.7%) reported challenges in accessing soap.

COVID-19, coronavirus disease 19; CI, confidence interval; SD, standard deviation; NA, Not available; 1y, primary outcome; 2y, secondary outcome.

The levels of handwashing have been presented as proportions of participants and health facilities that implemented handwashing practices as reported by the individual studies.

#### Levels of vaccine acceptance in Kenya and Uganda

**Figure 3:**
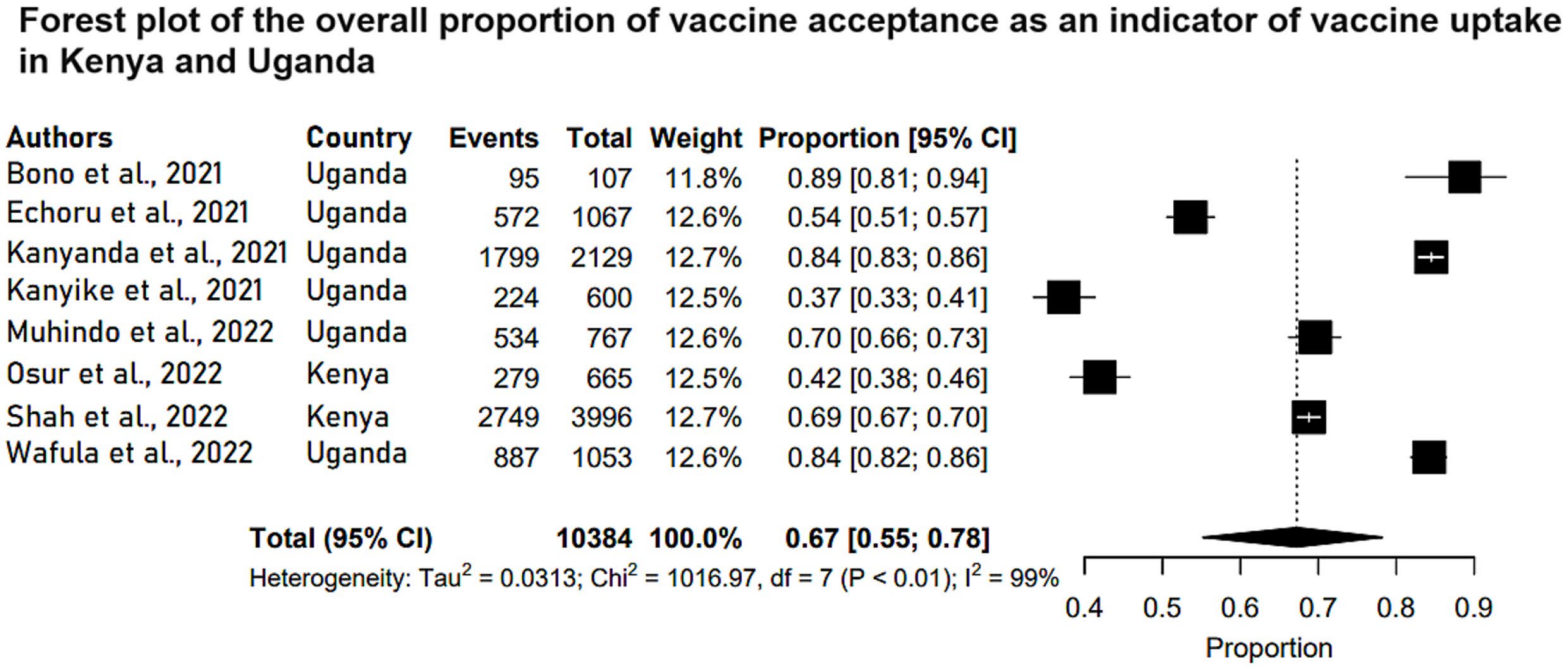
Forest plot of the overall proportion of vaccine acceptance as an indicator of vaccine uptake in Kenya and Uganda. Studies are plotted alphabetically. Each study is represented by a black box and a horizontal line, which correspond to the proportion and 95% confidence interval, respectively. *I*^2^ shows the degree of heterogeneity with value of *p* indicating whether there was statistically significance heterogeneity between the studies and among the groups.

On the other hand, 10 studies reported on levels of vaccine acceptance in Kenya and Uganda with Tanzania lacking a study with this outcome. Six of the 10 studies were conducted among Ugandan respondents. All the studies were conducted among participants 15 years old and above. The vaccine acceptance rate ranged between 37.3 and 84.5% across studies. Table 2 shows the characteristics of the studies that focused on vaccine acceptance to prevent and control COVID-19 in Kenya and Uganda. Meta-analysis of eight of those studies (*N* = 10,384) reported that the pooled estimate proportion for vaccine acceptance in Kenya and Uganda was 0.67 (95% CI: 0.55, 0.78) (Figure 3).

**Table 2:** Levels of vaccine acceptancy to prevent and control COVID-19 in Kenya and Uganda.

First author, year	Country	Participants	Age[mean(SD); range; median(IQR)]	Sample size (male)	Study design	Dates of data collection	Outcome	Outcome definition	Main findings
Kanyanda, 2021 (29)	Uganda	Respondents of national high-frequency phone surveys, aged 15 years and older, drawn from a nationally representative sample of households	≥15 years	2,129	Longitudinal high-frequency phone surveys	December 2020	Vaccine acceptancy (1y)	The proportion of participants willing to take COVID-19 vaccine.	Vaccine acceptance was estimated to be (84.5, 95% CI: 82.2 to 86.8%).
Echoru, 2021 (30)	Uganda	Adults of 18 to 70 years of age who had smartphones, and were capable of reading or using the Internet.	18–70 years	1,067 (781)	Cross-sectional study	July to September 2020	Vaccine acceptancy (1y)	Vaccine acceptance was defined as the proportion of participants willing to take COVID-19 vaccine.	The acceptance rate for vaccine acceptance intention against COVID-19 was (53.6%; 572/1067).
Bono, 2021 (31)	Uganda	Individuals 18 years and older who provided informed consent to participate in this study.	33.79 (8.84)years	107 (55)	Descriptive cross-sectional study	10 December 2020 to 9 February 2021	Vaccine acceptance (1y)	Vaccine acceptance was defined as the proportion of participants willing to take COVID-19 vaccine.	70 of 107 (65.4%) and 95 of 107 (88.8%) Ugandan participants were willing to take the COVID-19 vaccine at 90% effectiveness and 95% effectiveness, respectively.
Kanyike, 2021 (32)	Uganda	Medical students pursuing undergraduate degree programs of choice.	≥ 18 years	600 (377)	Online, descriptive, cross-sectional study using a quantitative approach.	Monday 15 March and Sunday 21 March 2021	Vaccine acceptance (1y)	Vaccine acceptance was defined as the proportion of participants willing to take COVID-19 vaccine.	37.3% (*n* = 224) of the participants were willing to take up the COVID-19 vaccine.
Wafula, 2022 (33)	Uganda	Adults 18 years and older with access to cell phones and who had been residents in the study district for at least 6 months.	34 (18–80) years	1,053 (651)	Nationwide cross-sectional survey	March 2021	Intention to vaccinate against COVID-19 (1y)	Vaccine acceptance was measured using a one-item question: ‘If a vaccine against COVID-19 becomes available, would you take it?’	Overall, 84.0% (887) of participants reported that they were likely to get the SARS-CoV-2 vaccine if it became available.
Osur, 2022 (34)	Kenya	Youths aged 18–35, registered in online platforms/peer groups that included Shujaaz, Brck Moja, Aifuence, Y Act and Heroes for Change.	18–35 years	665 (401 male)	Mixed-method study using a cross-sectional survey and focused group discussion approaches.	Not available	Vaccine acceptance (1y)	Percentage of participants willing to receive COVID-19 vaccine.	Only 42% of the youth were ready to be vaccinated.
Macharia, 2022 (35)	Kenya	Participants residing in Kenya, an African country, and Hungary a European country.	31.94(31.94) years	1,528	Cross-sectional study	April to August 2021	Vaccine acceptance rates (1y)	The proportion of the participants who were willing to receive the COVID-19 vaccine	Vaccine acceptance rates in Kenya were mean = 0.27, SD = 0.446, S.E = 0.045.
Muhindo, 2022 (36)	Uganda	People living with HIV ≥18 years seeking ART services regardless of the vaccination status who were able to speak English or Luganda (the local language in the area of Kampala).	(IQR) 36 (29–44) years	767 (282)	Cross-sectional survey	January to April 2022	Vaccine acceptability (1y)	Willingness to accept any of the available COVID-19 vaccines	Of the respondents, 534 (69.6, 95% confidence interval [CI]: 66.3–72.8%) reported receiving- at least one vaccine dose.
Muchiri, 2022 (37)	Kenya	Approved COVID-19 vaccination sites were downloaded from the Ministry of Health website.	> 18 years	622 vaccination sites	Mixed-method study	December 2021	Vaccine coverage (1y)	The proportion of the participants who had received the COVID-19 vaccine	At the national level, the COVID-19 vaccination coverage rate in December 2021 was 16.70% (95% CI: 16.66–16.74) – approx. 4.4 million but was lower in rural areas by 27.8%.
Shah, 2022 (38)	Kenya	The general adult public (patients and relatives) visiting the inpatient and outpatient clinics from six different healthcare facilities.	33 (26.5–43.0) years	3,996 (1789)	Cross-sectional survey	November 2021 and January 2022	Vaccine uptake levels (1y)	The proportion of the participants who had received the COVID-19 vaccine	Approximately 68.8% of the participants reported being vaccinated with at least one dose.

COVID-19, coronavirus disease 19; SARS-CoV-2, severe acute respiratory syndrome coronavirus-2; HIV, human immunodeficiency virus; CI, confidence interval; SD, standard deviation; IQR, inter-quartile range; S.E, standard error; 1y, primary outcome.

The levels of vaccine acceptancy have been presented as proportions of participants that were vaccinated or reported that they were likely to be vaccinated, and vaccination coverage rate in approved vaccination sites as reported by the individual studies.

#### Levels of vaccine hesitance in Kenya, Uganda and Tanzania

**Figure 4:**
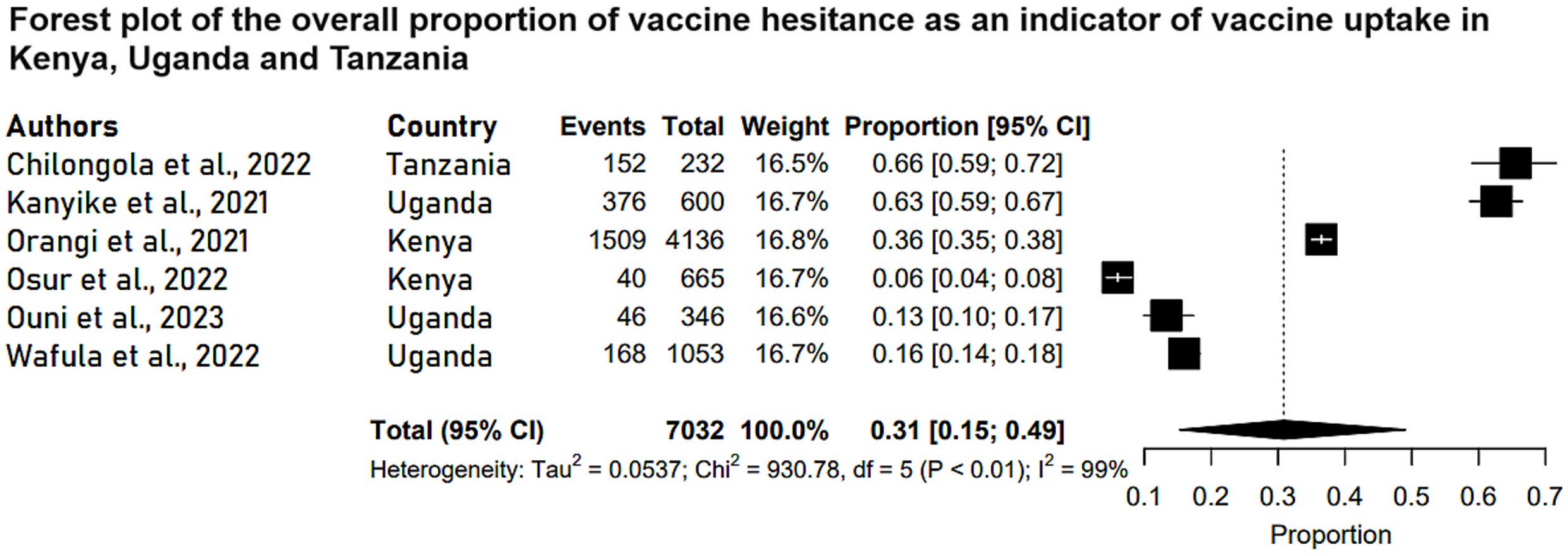
Forest plot of the overall proportion of vaccine hesitance as an indicator of vaccine uptake in Kenya, Uganda and Tanzania. Each study is represented by a black box and a horizontal line, which correspond to the proportion and 95% confidence interval, respectively. *I*^2^ shows the degree of heterogeneity with value of *p* indicating whether there was statistically significance heterogeneity between the studies and among the groups.

Six observational studies reported the levels of COVID-19 vaccine hesitance among the study participants in Kenya, Uganda and Tanzania. These studies were conducted among adult respondents above 18 years of age. Three of the studies reported vaccine hesitance as a secondary outcome indicating vaccine uptake levels in the three countries. The COVID-19 vaccine hesitance levels across studies were between 6 and 65.5% among the study participants. The characteristics of studies of vaccine hesitance in Kenya, Uganda and Tanzania are depicted in Table 3. A meta-analysis of the six studies (N = 7,032) reported that the pooled estimate proportion was 0.31 (95% CI: 0.15, 0.49) in Kenya, Uganda and Tanzania (Figure 4).

**Table 3:** Levels of vaccine hesitancy to prevent and control COVID-19 in Kenya, Uganda and Tanzania.

First author, year	Country	Participants	Age [mean(SD); range; median(IQR)]	Sample size (male)	Study design	Dates of data collection	Outcome	Outcome definition	Main findings
Chilongola, 2022 (39)	Tanzania	Individuals who visited their relatives who were admitted or undergoing medical care at Kilimanjaro Christian Medical Centre were requested to respond to structured questions regarding COVID-19.	33 (25–45) years	232 (168)	Cross-sectional study	October 2021	Vaccine hesitancy (1y)	The proportion of participants who were unwilling to take COVID-19 vaccine	152 (65.52%) of interviewed participants had a negative attitude toward COVID-19 vaccines.
Kanyike, 2021 (32)	Uganda	Medical students pursuing undergraduate degree programs of choice.	≥18 years	600 (377)	Cross-sectional study	Monday 15 March and Sunday 21 March 2021	Vaccine hesitancy (2y)	Vaccine hesitancy was defined as the proportion of individuals reluctant to take COVID-19 vaccine.	The majority of the participants (*n* = 376, 62.7%) were not willing to be vaccinated against COVID-19.
Wafula, 2022 (33)	Uganda	Adults 18 years and older with access to cell phones and who had been residents in the study district for at least 6 months.	34 (18–80) years	1,053 (651)	Cross-sectional survey	March 2021	Intention to vaccinate against COVID-19 (2y)	Intention to take the COVID-19 vaccine was measured using a one-item question: ‘If a vaccine against COVID-19 becomes available, would you take it?’	Overall, 16.0% (168) of the participants reported that they would not get the SARS-CoV-2 vaccine if it became available.
Ouni, 2023 (40)	Uganda	Registered and practising health workers in the Dokolo district from both government and private health facilities.	31.4 (6.9) years	346 (151)	Mixed-method, cross-sectional descriptive study	Not available	Vaccine hesitancy (1y)	Vaccine hesitancy was defined as the unwillingness of a health worker to take a COVID-19 vaccine	Of the 346 health workers enrolled, (13.3% [46/346]) were vaccine-hesitant.
Osur, 2022 (34)	Kenya	Youths aged 18–35, registered in online platforms/peer groups that included Shujaaz, Brck Moja, Aifuence, Y Act and Heroes for Change.	range 18–35 years	665 (401)	Mixed-method study using a cross-sectional survey and focused group discussions approaches.	Not available	Vaccine hesitancy (2y)	Percentage of participants unwilling to receive COVID-19 vaccine.	6% of the participants were unwilling to be vaccinated.
Orangi, 2021 (41)	Kenya	Participants were sampled from households in four existing Population Council prospective cohort studies across four counties: Kilifi, Kisumu, Nairobi and Wajir.	40.8 (12.6) years	4,136 (1355)	Cross-sectional study	February 2021	Levels of vaccine hesitancy (1y)	Percentage of participants unwilling to receive COVID-19 vaccine.	Overall, the level of vaccine hesitancy towards the COVID-19 vaccine across all study counties was 36% (n = 1,509).

COVID-19, coronavirus disease 19; SARS-CoV-2, severe acute respiratory syndrome coronavirus-2; CI, confidence interval; SD, standard deviation; IQR, inter-quartile range; S.E, standard error; 1y, primary outcome; 2y, secondary outcome.

The levels of vaccine hesitancy have been presented as proportions of participants that were unwilling to get vaccinated against COVID-19 as reported by the individual studies.

### Publication bias

A funnel plot was evaluated for all three outcomes and the results are shown on Supplementary Figures 1–3. The two sides of each funnel plot were symmetrical, and no significant publication bias was found in this study.

### Heterogeneity

The meta-analyzed studies revealed substantial heterogeneity, with *I*^2^ statistics indicating 98% for handwashing levels, 99% for vaccine acceptance levels, and 99% for vaccine hesitance levels, as depicted in Figures 1–3, respectively.

### Qualitative analysis

#### Levels of handwashing in Kenya, Uganda and Tanzania

The participants of the studies that reported handwashing levels as a way of preventing and controlling COVID-19 were adults with a mean (sd) ages ranging from 34.8(11.2) years to 38.2(14.8) years. These studies reported that approximately 80.4 to 97% of the respondents practiced handwashing and 28.6% of facilities enforced obligatory use of hand hygiene (Table 1).

#### Levels of vaccine acceptance in Kenya and Uganda

On the other hand, 10 studies reported on levels of vaccine acceptance in Kenya and Uganda with Tanzania lacking a study with this outcome. Six of the 10 studies were conducted among Ugandan respondents. All the studies were conducted among participants 15 years old and above. The vaccine acceptance rate ranged between 37.3 and 84.5% across studies. Table 2 shows the characteristics of the studies that focused on vaccine acceptance to prevent and control COVID-19 in Kenya and Uganda.

#### Levels of vaccine hesitancy in Kenya, Uganda and Tanzania

Six observational studies reported the levels of COVID-19 vaccine hesitance among the study participants in Kenya, Uganda and Tanzania. These studies were conducted among adult respondents above 18 years of age. Three of the studies reported vaccine hesitancy as a secondary outcome indicating vaccine uptake levels in the three countries. The COVID-19 vaccine hesitancy levels across studies were between 6 and 65.5%mong the study participants. The characteristics of studies of vaccine hesitance in Kenya, Uganda and Tanzania are depicted in Table 3.

## Discussion

To date, there are scarce data on the levels of handwashing and COVID-19 vaccine uptake in East Africa. Information regarding these interventions to contain the spread of SARS-CoV-2 is helpful in developing public health strategies of managing COVID-19. This study involved conducting a systematic review and meta-analysis of handwashing and vaccine uptake levels among Kenyan, Ugandan, and Tanzanian study participants.

In the current study, a total of 17 articles were systematically reviewed after which 14 were included in the final analysis conducted according to the outcome measure. Overall, the included studies had high methodological quality and the pooled handwashing levels in Kenya, Uganda and Tanzania were 88% (95% CI: 73, 97). The level of vaccine uptake in the three countries was fairly high as indicated by vaccine acceptance and vaccine hesitance at 67% (95% CI: 55, 78) and 31% (95% CI: 15, 49), respectively.

Handwashing was associated with reduced levels of COVID-19 suggesting that this strategy could be effective in the control and prevention of COVID-19. These findings are concordant with those reported by Beale et al. showing that handwashing (6–10 times per day) predicted a decreased susceptibility to coronavirus infection (13).

Despite clinical trials reporting most COVID-19 vaccines to be harmless and efficacious (42), the findings of this study suggest that there are considerable levels of vaccine hesitance in Kenya, Uganda and Tanzania. These findings are concordant with those of Afolabi and Ilesanmi, and Mutombo et al. (43, 44) which reported considerable vaccine hesitancy in Africa in addition to low COVID-19 vaccine coverage in the continent.

In our meta-analyses, we acknowledge the presence of substantial heterogeneity, with *I*^2^ statistics ranging from 98 to 99%. This observation warrants careful consideration, as it underscores the need for a nuanced interpretation of our findings. The sources of this heterogeneity are multifaceted. One contributing factor could be the inherent regional differences within the study populations, spanning Kenya, Uganda, and Tanzania. Additionally, variability in outcome definitions employed across the studies may have added to this heterogeneity. Therefore, the results of this systematic review and meta-analysis should be interpreted with caution.

To the best of our knowledge, this is the first systematic review and meta-analysis exploring levels of handwashing and vaccine uptake in the prevention and control of COVID-19 in Kenya, Uganda and Tanzania. Moreover, our study is comprehensive as it includes a large number of relevant articles published to date. Our systematic review and meta-analysis has a few limitations: we may have missed some studies since some African journals are not indexed in PubMed. Lastly, only observational studies, which tend to provide weaker evidence compared to randomized clinical trials, were included in the meta-analysis section and some studies could not be meta-analyzed.

## Conclusion

Evidence of the levels of handwashing and vaccine uptake in Kenya, Uganda and Tanzania is limited and inconsistent. Leaders should champion awareness and COVID-19 vaccine uptake and improve handwashing facilities. Our findings warrant further investigation to determine the levels of handwashing and vaccine uptake to control and prevent COVID-19.

## Data availability statement

The original contributions presented in the study are included in the article/Supplementary material, further inquiries can be directed to the corresponding author.

## Author contributions

JoM: Conceptualization, Data curation, Formal analysis, Funding acquisition, Investigation, Methodology, Project administration, Supervision, Validation, Visualization, Writing – original draft, Writing – review & editing. JaM: Data curation, Methodology, Writing – review & editing. HM: Data curation, Methodology, Writing – review & editing. KK: Data curation, Methodology, Writing – review & editing. SK: Data curation, Methodology, Writing – review & editing. LK: Data curation, Methodology, Writing – review & editing. RM: Data curation, Methodology, Writing – review & editing. MN: Data curation, Methodology, Writing – review & editing.

## Footnotes

^1^ https://www.nhlbi.nih.gov/health-topics/study-quality-assessment-tools
