# Supplementary material for: Levels of handwashing and vaccine uptake in Kenya, Uganda, and Tanzania to prevent and control COVID-19: a systematic review and meta-analysis
Source: Front Public Health. 2023 Nov 9;11:1256007. doi: 10.3389/fpubh.2023.1256007 (PMC10666047; doi:10.3389/fpubh.2023.1256007)
Supplement: Supplementary file 1 [file Data_Sheet_1.docx]

Supplementary Material

# Supplementary Figures

**Supplementary figure 1**


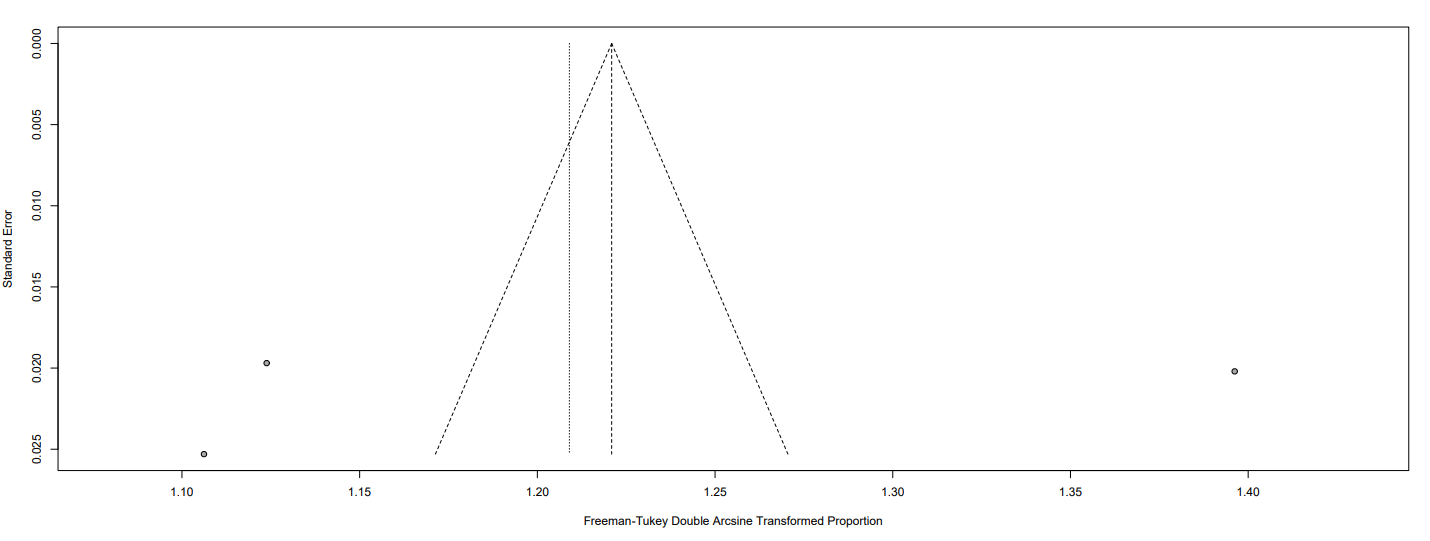


Supplementary figure 1. Funnel plot to assess publication bias among studies evaluating handwashing in Kenya, Uganda and Tanzania. The triangle represents the estimates of the included studies that reported on levels of handwashing. The Freeman-Tukey double arcsine transformed is plotted on the horizontal axis, against the standard error of the transformed proportion. The vertical line in the funnel plot indicates the random effect summary estimate and the sloping two lines indicate the expected 95% confidence intervals for a given standard error.

**Supplementary figure 2**


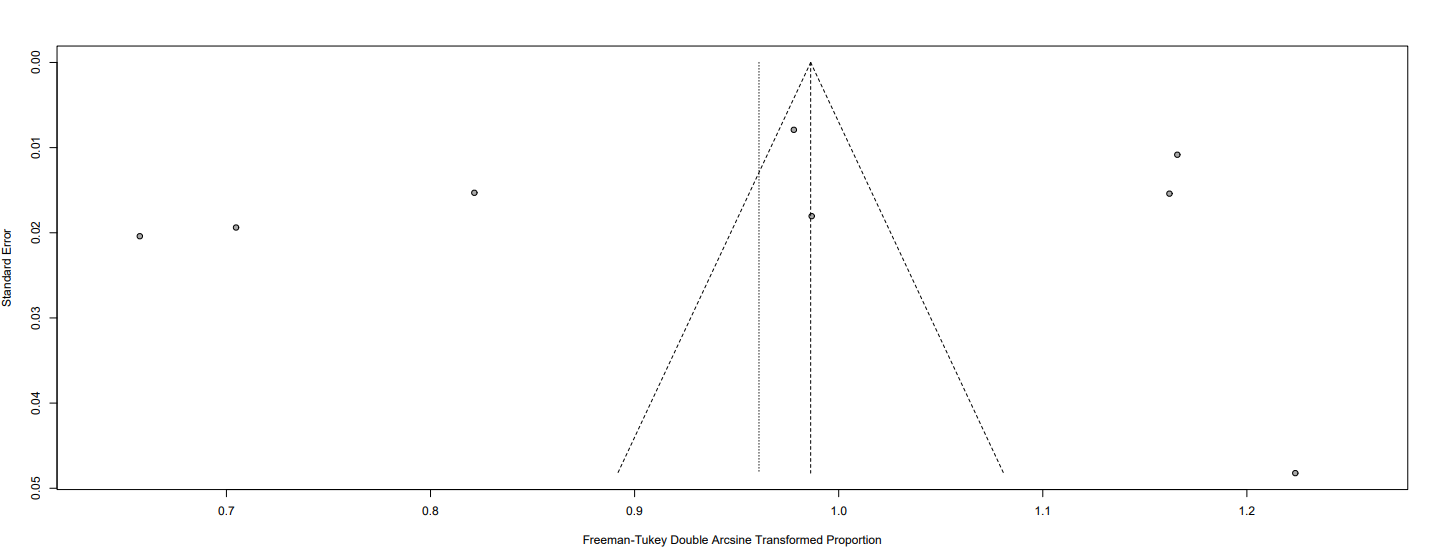


Supplementary figure 2. Funnel plot to assess publication bias among studies evaluating vaccine acceptance in Kenya and Uganda. The triangle represents the estimates of the included studies that reported on vaccine acceptance. The Freeman-Tukey double arcsine transformed is plotted on the horizontal axis, against the standard error of the transformed proportion. The vertical line in the funnel plot indicates the random effect summary estimate and the sloping two lines indicate the expected 95% confidence intervals for a given standard error.

**Supplementary figure 3**


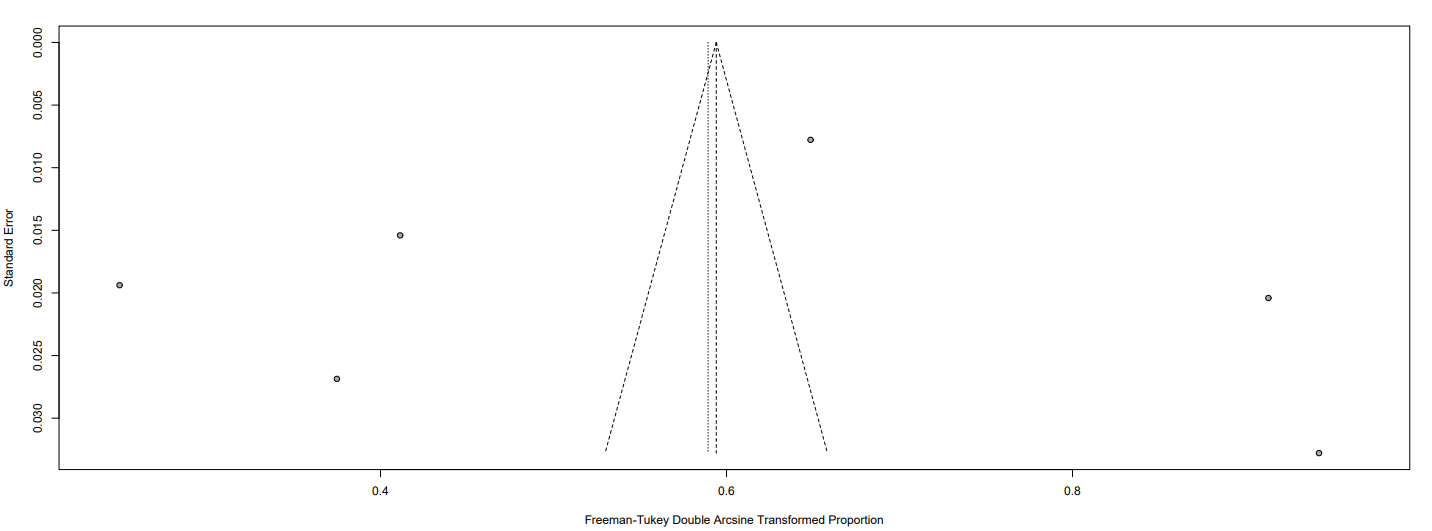


Supplementary figure 3. Funnel plot to assess publication bias among studies evaluating vaccine hesitance in Kenya, Uganda and Tanzania. The triangle represents the estimates of the included studies that reported on vaccine hesitance. The Freeman-Tukey double arcsine transformed is plotted on the horizontal axis, against the standard error of the transformed proportion. The vertical line in the funnel plot indicates the random effect summary estimate and the sloping two lines indicate the expected 95% confidence intervals for a given standard error.

# Supplementary Tables

| Supplementary table 1. Search terms and their keywords used to formulate medical subject heading (MeSH) terms. | | |
| --- | --- | --- |
| **Term** | **Key words** | **MeSH terms** |
| Handwashing | Handwashing  Hand hygiene | ("hand disinfection"[MeSH Terms] OR Handwashing[Text Word] OR "hand hygiene"[MeSH Terms] OR hand hygiene[Text Word]) |
| Vaccination | Vaccine  Vaccination  Immunization | ("vaccination"[MeSH Terms] OR vaccination[Text Word] OR "immunization"[MeSH Terms] OR immunization[Text Word]) |
| COVID-19 | COVID-19  SARS-CoV2 | ("COVID-19"[All Fields] OR "COVID-19"[MeSH Terms] OR "SARS-CoV-2"[All Fields] OR "sars-cov-2"[MeSH Terms] OR "Severe Acute Respiratory Syndrome Coronavirus 2"[All Fields]) |
| Prevention | Prevention  Control | ("prevention and control"[Subheading] OR prevention[Text Word] OR "control groups"[MeSH Terms] OR control[Text Word]) |
| Policy | Policy | ("policy"[MeSH Terms] OR policy[Text Word]) |
| Kenya | Kenya | ("Kenya"[MeSH Terms] OR Kenya[Text Word]) |
| Uganda | Uganda | ("Uganda"[MeSH Terms] OR Uganda[Text Word]) |
| Tanzania | Tanzania | ("Tanzania"[MeSH Terms] OR Tanzania[Text Word]) |
| **Abbreviations and acronyms:** COVID-19, Coronavirus disease 2019; SARS-CoV2, severe acute respiratory syndrome coronavirus 2; MeSH, Medical subject heading.  The keywords were derived from the research questions and were used to identify MeSH terms on PubMed database. | | |

| Supplementary table 2. Search strategy used to identify the relevant papers published on PubMed database. | | | |
| --- | --- | --- | --- |
| **Database** | **Date searched** |  | **Search strategy** |
| **PubMed** | 17/01/2022 | (((term 1) OR (term 2) AND (term 3) AND (term 4) AND (term 5)) AND ((term 6) OR (term 7) OR (term 8))) | ((("hand disinfection"[MeSH Terms] OR Handwashing[Text Word] OR "hand hygiene"[MeSH Terms] OR hand hygiene[Text Word]) OR ("vaccination"[MeSH Terms] OR vaccination[Text Word] OR "immunization"[MeSH Terms] OR immunization[Text Word]) AND ("COVID-19"[All Fields] OR "COVID-19"[MeSH Terms] OR "SARS-CoV-2"[All Fields] OR "sars-cov-2"[MeSH Terms] OR "Severe Acute Respiratory Syndrome Coronavirus 2"[All Fields]) AND ("prevention and control"[Subheading] OR prevention[Text Word] OR "control groups"[MeSH Terms] OR control[Text Word]) AND ("policy"[MeSH Terms] OR policy[Text Word])) AND (("Kenya"[MeSH Terms] OR Kenya[Text Word]) OR ("Uganda"[MeSH Terms] OR Uganda[Text Word]) OR ("Tanzania"[MeSH Terms] OR Tanzania[Text Word]))) |
| **Abbreviations and acronyms:** COVID-19, Coronavirus disease 2019; SARS-CoV2, severe acute respiratory syndrome coronavirus 2; MeSH, Medical subject heading.  The search strategy was created by merging the keywords: handwashing, vaccine, COVID-19, prevention, policy, Kenya, Uganda, and Tanzania, which was later customized to suit various databases. | | | |

Supplementary table 3. Quality Assessment Tool for Observational Cohort and Cross-sectional studies

|  | **Chilongola, 2022** | **Mghamba, 2022** | **Kanyanda, 2021** | **Echoru, 2021** | **Bono, 2021** | **Kanyike, 2021** | **Mboowa, 2021** | **Wafula, 2022** | **Ouni, 2023** | **Okedi,2022** | **Mwai, 2022** | **Osur, 2022** | **Macharia, 2022** | **Orangi, 2021** | **Muhindo, 2022** | **Muchiri, 2022** | **Shah, 2022** |
| --- | --- | --- | --- | --- | --- | --- | --- | --- | --- | --- | --- | --- | --- | --- | --- | --- | --- |
| Was the research question or objective clearly stated? | YES | YES | YES | YES | YES | YES | YES | YES | YES | YES | YES | YES | YES | YES | YES | YES | YES |
| Was the study population clearly specified and defined? | YES | YES | YES | YES | YES | YES | YES | YES | YES | YES | YES | YES | YES | YES | YES | YES | YES |
| Was the participation rate of eligible persons at least 50%? | YES | YES | YES | YES | YES | YES | YES | YES | YES | YES | YES | YES | YES | YES | YES | YES | YES |
| Were all the subjects selected or recruited from the same or similar populations (including the same time period)? Were inclusion and exclusion criteria for being in the study pre-specified and applied uniformly to all participants? | YES | YES | YES | YES | YES | YES | YES | YES | YES | YES | YES | YES | YES | YES | YES | YES | YES |
| Was a sample size justification, power description, or variance and effect estimates provided? | YES | YES | YES | YES | NO | YES | YES | YES | YES | YES | YES | YES | YES | YES | YES | YES | NO |
| For the analyses in this paper, were the exposure(s) of interest measured prior to the outcome(s) being measured? | YES | YES | YES | YES | YES | YES | YES | YES | YES | YES | YES | YES | YES | YES | YES | YES | YES |
| Was the timeframe sufficient so that one could reasonably expect to see an association between exposure and outcome if it existed? | YES | YES | YES | YES | YES | YES | YES | YES | YES | YES | YES | YES | YES | YES | YES | YES | YES |
| For exposures that can vary in amount or level, did the study examine different levels of the exposure as related to the outcome (e.g., categories of exposure, or exposure measured as continuous variable)? | YES | NA | NA | NA | NA | NA | NA | NA | NA | NA | NA | NA | NA | NA | NA | NA | NA |
| Were the exposure measures (independent variables) clearly defined, valid, reliable, and implemented consistently across all study participants? | YES | YES | YES | YES | YES | YES | YES | YES | YES | YES | YES | YES | YES | YES | YES | YES | YES |
| Was the exposure(s) assessed more than once over time? | NA | NA | NA | NA | NA | NA | NA | NA | NA | NA | NA | NA | NA | NA | NA | NA | NA |
| Were the outcome measures (dependent variables) clearly defined, valid, reliable, and implemented consistently across all study participants? | YES | YES | YES | YES | YES | YES | YES | YES | YES | YES | YES | YES | YES | YES | YES | YES | YES |
| Were the outcome assessors blinded to the exposure status of participants? | NA | NA | NA | NA | NA | NA | NA | NA | NA | NA | NA | NA | NA | NA | NA | NA | NA |
| Was loss to follow-up after baseline 20% or less? | NA | NA | NA | NA | NA | NA | NA | NA | NA | NA | NA | NA | NA | NA | NA | NA | NA |
| Were key potential confounding variables measured and adjusted statistically for their impact on the relationship between exposure(s) and outcome(s)? | NR | NR | NR | NR | NR | NR | YES | YES | NR | NR | NR | NR | NR | NR | NR | NR | NR |
|  |  |  |  |  |  |  |  |  |  |  |  |  |  |  |  |  |  |
| *CD, cannot determine; NA, non applicable; NR, not registered |  |  |  |  |  |  |  |  |  |  |  |  |  |  |  |  |  |
